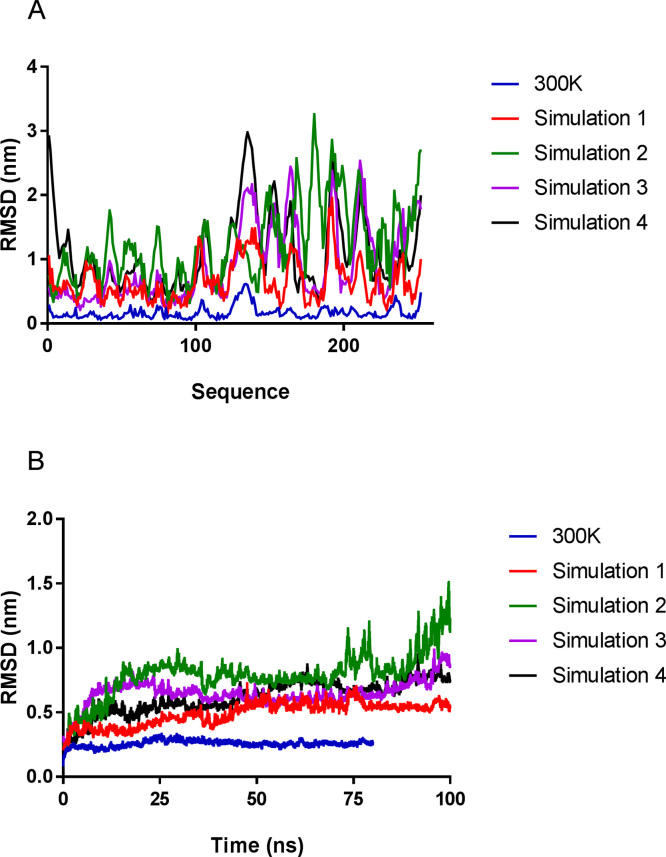# Identification of B cell epitopes enhanced by protein unfolding and aggregation

**DOI:** 10.1016/j.molimm.2018.11.020

**Published:** 2019-01

**Authors:** Timothy J. Eyes, James I. Austerberry, Rebecca J. Dearman, Linus O. Johannissen, Ian Kimber, Noel Smith, Angela Thistlethwaite, Jeremy P. Derrick

**Affiliations:** a Lydia Becker Institute of Immunology and Inflammation, School of Biological Sciences, Faculty of Biology, Medicine and Health, Manchester Academic Health Science Centre, The University of Manchester, Oxford Road, Manchester, UK; b Faculty of Science and Engineering, Manchester Institute of Biotechnology, The University of Manchester, Princess Street, Manchester, UK; c Lonza, Granta Park, Cambridge, UK

**Keywords:** ADA, anti-drug antibody, APC, antigen processing cell, BSA, bovine serum albumin, CD, circular dichroism, CDR, complementarity determining region, DEAE, diethylaminoethyl, DLS, dynamic light scattering, ELISA, enzyme linked immunosorbent assay, Fmoc, fluorenylmethyloxycarbonyl, FTIR, Fourier-transform infrared, HRP, horse radish peroxidase, i.p., intraperitoneal, IPTG, β-D-1-thiogalactopyranoside, Ig, immunoglobulin, LPS, lipopolysaccharide, MD, molecular dynamics, MHC, major histocompatibility complex, OD, optical density, PAMP, pathogen associated molecular pattern, PBMC, peripheral blood mononuclear cell, PBS, phosphate buffered saline, RH, hydrodynamic radius, RMSD, root mean square deviation, RMSF, root mean square fluctuation, scFv, single chain variable fragment, TPP, therapeutic protein product, Ti, T cell independent, Td, T cell dependent, TLR, toll-like receptor, TNF, tumour necrosis factor, B cell epitope, Aggregation, Biopharmaceutical, Immunogenicity

## Abstract

Aggregation of therapeutic proteins is a key factor in the generation of unwanted immunogenicity, and can result in reduced serum half-life, neutralization of function and adverse health effects. There is currently little information regarding how aggregates interact with B-cell receptors or cognate antibodies at the protein sequence level, or whether non-native, aggregate-induced epitopes predominate in these interactions. Using an antibody fragment (single chain antibody variable fragment; scFv) that forms aggregates readily at low temperature, anti-scFv IgG antibody responses were generated by intraperitoneal injection of BALB/c strain mice with monomer or aggregate preparations. Aggregate-specific immunosignatures were identified by oligo-peptide microarray fine epitope mapping, using overlapping 15mer peptides based on the linear sequence of scFv, printed onto glass slides. IgG antibodies from mice immunized with aggregated scFv preferentially recognized a patch of overlapping peptides. This region mapped to a β-strand located at the interface between the V_H_ and V_L_ domains. Molecular dynamics simulations indicated that the V_L_ domain is less stable than the V_H_ domain, suggesting the interface region between the two domains becomes exposed during partial unfolding of the scFv during aggregate formation. These data are consistent with the hypothesis that epitopes from partially unfolded states are revealed, or are more fully exposed, in the aggregated state, and that this can augment the IgG antibody response. This observation offers the theoretical possibility that epitopes preferentially associated with aggregates can be identified from the anti-drug antibody serum IgG response which may, in turn, lead to better methods for detection of anti-drug antibody responses, and improved design of therapeutic proteins to control immunogenicity.

## Highlights

• Aggregation of an exemplar therapeutic antibody fragment (scFv) enhances immunogenicity *in vivo*.
• Epitope mapping reveals immunogenicity is directed to a specific epitope in aggregate species.
• Molecular simulation demonstrates biophysical stress enhances epitope presentation.
• Protein aggregates have distinct immunological profiles to their native counterparts.

## 1 Introduction

Biotherapeutic proteins are in extensive clinical use and cover a range of pharmaceutical products, from vaccines to hormones, cytokines and monoclonal antibodies. They are used to treat a wide range of conditions, including malignant and inflammatory diseases, and they now comprise a substantial share of the global drugs market (Chan and Carter, 2010). A major consideration, which influences both efficacy and safety, is the potential for the development of anti-drug immune responses in the patient. This is in spite of the fact that many therapeutic proteins, particularly monoclonal antibodies, are derived from human germline sequences, and should therefore, in theory, be immunologically inert.

Anti-drug antibodies (ADAs) can be categorized as non-neutralizing or neutralizing, depending on their ability to impair therapeutic function. In some instances ADA responses can lead to adverse health effects, the most prominent examples being the use of recombinant erythropoietin leading to pure red cell aplasia, and the withdrawal of Factor VIII products from haemophilia patients (Yin et al., 2015). Therapeutic monoclonal antibodies are frequently administered at high concentrations (above 50 mg/ml), one consequence of this being that they frequently form aggregates (irregular, multimeric assemblies covering a wide range of sizes, from nm up to visible particles) (Ratanji et al., 2014). Aggregation is a term which describes the association of protein monomers into assemblies that are not defined, quaternary structures. Protein aggregates can form linear, ordered assemblies (e.g. amyloid), or disordered structures (Ratanji et al., 2014). In the case of therapeutic proteins, aggregates generally fall into the latter category. They span a wide range of sizes, from a few nm up to microns in dimension. Large sub-visible and visible particles are often insoluble, and their formation can complicate and obstruct bioprocessing- by blocking filters, for example, and reducing overall yield. Aggregates can form on storage, limiting the shelf life of a protein drug (Mahler et al., 2009). A substantial body of evidence, from clinical studies, *in vivo* animal models, and *ex vivo* experiments, has established that TPP (therapeutic protein product) aggregation is a major risk factor for immunogenicity and plays a key role in breaking immunological tolerance (Bessa et al., 2015; Boll et al., 2017; Filipe et al., 2010; Foged and Sundblad, 2008; Moussa et al., 2016; Ratanji et al., 2014; Rosenberg, 2006; Yin et al., 2015). Our recent work has shown that aggregation is associated with a skewing of immune responses towards a selective T helper 1 (Th1)-type phenotype (Ratanji et al., 2016a).

While it is now clear that aggregation can enhance immunogenicity and modify the quality and vigor of the adaptive immune response, the exact mechanisms through which aggregates bypass immunological tolerance and provoke immunogenicity are not well understood, although recent evidence suggests that both T cell-dependent and T cell- independent mechanisms are important (Moussa et al., 2016). Protein aggregates are thought to enhance immune stimulation through several intrinsic properties (Filipe et al., 2010). Two parallel cellular pathways exist through which B cells are activated by aggregates and produce antibodies against the antigen: the T cell-dependent and T cell-independent pathways (Td and Ti, respectively) (Foged and Sundblad, 2008). In the Td pathway, aggregate is first endocytosed and processed by resident antigen presenting cells (APC). Antigenic peptides are presented by the APC through major histocompatibility complex class II (MHC II) to cognate CD4^+^ helper T-cells which are able to activate a corresponding B-cell and stimulate antibody production to the protein aggregate (Foged and Sundblad, 2008). Relationships between TPP aggregate size and other factors (e.g. glycosylation), and specific antigen uptake and processing pathways are unclear at present. Indeed, the dimensions of sub-visible TPP particles lie close to those of a single cell (∼10 μm), indicating that the uptake pathway differs from monomeric, soluble TPP antigen (Filipe et al., 2010). Unlike monomeric protein, aggregate species may be able to stimulate antigen-presenting dendritic cells (DC) through binding by Toll-like(TLR) and Fc receptors (Rombach-Riegraf et al., 2014). The greater molecular size of aggregate particles means that there is an elevated antigen load, increasing the quantity and altering the profile of MHC associated epitopes by mass action (Rombach-Riegraf et al., 2014). In the Ti pathway, aggregates are able to stimulate antibody production through crosslinking of B-cell receptors on the cell surface, independently of T cell help and APCs (Ratanji et al., 2014). We have also identified that aggregates may associate with host expression cell impurities and that this can provide additional drivers for immunogenicity (Ratanji et al., 2016b).

Current models of aggregate formation suggest that they are created by association of aggregation-prone but native-like states, which act as nuclei for growth (Mahler et al., 2009). Aggregates, by their nature, are heterogeneous species: they only partially resemble the original molecule, possessing novel structures and patterns that may also modulate their immunogenic potential. Neo-epitopes may be generated by monomer association. Higher order structures and the formation of repetitive molecular patterns are thought to be key drivers of immune recognition, as they resemble microbial patterns (PAMP-like pathogen associated molecular patterns) to which the immune system is adapted to respond (Filipe et al., 2010). Aggregates generated through biophysical stress are likely to be composed of partially unfolded or fully denatured monomers which may also lead to the exposure of cryptic epitopes, normally buried within the native structure (Austerberry et al., 2017). To date, these kinds of cryptic epitopes have been postulated but not characterized at the molecular or epitope level (Filipe et al., 2010; Van Regenmortel, 2009).

One approach to the study of altered immune responses associated with aggregation is the use of immunoprofiling to compare antibody responses to both monomer and aggregates for a protein which is typical of the class used for clinical purposes (e.g. an immunoglobulin). Immunoprofiling uses a peptide microarray to identify patterns of antibody recognition profiles, and thereby provide an immunological ‘fingerprint’ of serum antibody responses against a defined library of peptide antigens (Legutki et al., 2014). This method has been used in vaccine studies, for example, to examine the maturation of antibody responses and to measure elevated antibody responses to particular epitopes in protected individuals (Legutki and Johnston, 2013). Immunoprofiling is now also emerging as a tool for profiling patient immunogenicity to therapeutic antibodies. Homann et al. used a peptide array to characterize patient ADA epitope profiles to the anti-tumor necrosis factor (TNF) therapeutic monoclonal antibodies Infliximab and Adalimumab, drugs which have known immunogenicity (Homann et al., 2015, 2017). Immune signatures were successfully identified in patient sera that indicated ADAs binding to a number of epitope sequences in the variable region of the parent therapeutic antibody.

Here we describe the use of a custom peptide microarray to identify, for the first time, an epitope associated with the aggregation of a scFv format antibody, studied as an exemplar biopharmaceutical protein. The data indicate that the induction of an immune response to a specific region of the scFv revealed by partial denaturation occurs only on exposure to the aggregate. The results therefore demonstrate that it is indeed possible to identify epitopes enhanced by aggregation from serum IgG responses, suggesting a novel approach that could be applied to clinical samples to determine whether aggregation-specific ADAs pose a particular immunogenicity safety issue.

## 2 Materials and methods

### 2.1 scFv production

A humanized anti c-Met scFv (Edwardraja et al., 2010) was produced as previously described (Ratanji et al., 2016a). In brief, scFv was cloned into the pET-22b vector (Novagen) and expressed in ClearColi® BL21 (DE3) *E. coli* cells (Lucigen, USA). Transformants were cultured at 30 °C to an optical density (OD) of 0.8 at 600 nm, induced with isopropyl β-D-1-thiogalactopyranoside (IPTG) and incubated overnight at 16 °C. Cell pellets were resuspended, sonicated and centrifuged at 28,672 *g* for 30 min. scFv was purified from supernatants using DEAE (diethylaminoethanol) Sepharose anion exchange chromatography, followed by Protein A affinity and size exclusion chromatography.

### 2.2 Endotoxin testing

ClearColi® is an *E.coli* strain which produces a modified lipopolysaccharide (LPS) that does not induce endotoxin responses in mammalian cells, thus reducing pyrogenic stimulation by LPS contaminants remaining within recombinant protein preparations during *in vivo* exposure (Mamat et al., 2015). After purification, recombinant scFv protein was tested for active endotoxin content by using a HEKblue human TLR4 reporter cell line, according to the suppliers’ specifications (KB-hTLR4, Invivogen, USA). In brief, HEKblue cells were cultured in DMEM medium with 50U/ml penicillin, 50 μg/ml streptomycin, 100 μg/mL Normocin and 2 mM l-glutamine, and grown to 80% confluency at 37 °C and 5% humidity. Cells were resuspended in Dulbecco’s phosphate buffered saline (PBS) and diluted to 1.4 × 10^5^ cells/mL in detection medium containing alkaline phosphatase substrate (Invivogen, USA). Cells were then transferred to flat bottomed 96 well tissue culture plates containing test protein, controls and LPS standards, and then incubated for 6 h at 37**°**C and 5% humidity. Reporter gene activity was determined by measuring absorbance at 650 nm filter using an automated multiwell plate reader (ELx800, BioTek, USA). Test protein was deemed free of active LPS if the absorbance values for 1 mg/mL were less than the mean of the PBS control plus three times standard deviation.

### 2.3 Generation of scFv aggregates

Purified monomeric scFv was diluted to 1 mg/mL in 1 mL of Dulbeccos PBS without Ca^2+^ or Mg^2+^ (Sigma Aldrich, USA) and stressed by heating at 40 °C for 25 min in a static heating block.

### 2.4 Animal experiments

Young adult (8–12 weeks old) female BALB/c strain mice were used for these experiments (Envigo, UK). Mice were housed on sterilized wood bedding with materials provided for environmental enrichment. Food (Beekay Rat and Mouse Diet No1 pellets; B&K Universal, UK) and water were available *ad libitum*. The ambient temperature was maintained at 21 +/- 2 °C and relative humidity was 55 +/- 10% with a 12 h light/dark cycle. All procedures were carried out in accordance with the Animals (Scientific Procedures) Act 1986, and approved by Home Office licence. Mice were immunized by intraperitoneal (i.p.) injection with 250 μL of 1.0 mg/mL protein (monomeric or aggregate) in PBS on days 0, 7, 14 and exsanguinated on day 21. Serum was isolated from whole blood for evaluation and stored at −80 °C until analysis. Negative control mice were untreated (naïve).

### 2.5 ELISA for protein and peptide specific antibody classes and subclasses

For protein antigen detection, plastic Maxisorb plates (Nunc) were coated with 10 μg/mL of protein in PBS overnight at 4 °C. Plates were blocked with 2% (w/v) bovine serum albumin (BSA)/PBS (Sigma Aldrich) at 37 °C for 30 min. For peptide antigen, Neutravidin-coated plates (pre-blocked with Superblock, Thermo, USA) were coated with 10 μg/mL biotinylated peptide (Pepceuticals, UK) in PBS overnight at 4 °C. Doubling dilutions of serum samples were added in duplicate (starting dilution 1:32 or 1:64 for anti-IgG; 1:128 or 1:64 for anti IgM antibody analyses) in 1% BSA/ PBS (as a negative control naive mouse serum samples was added to plates) and incubated for 3 h at 4 °C. Plates were incubated for 2 h at 4 °C with horseradish peroxidise (HRP) labelled sheep anti-mouse IgG diluted 1:4000, sheep anti-mouse IgG1 HRP diluted 1:2000, sheep anti-mouse IgG2a HRP diluted 1:1000 (all BioRad, USA) or goat anti-mouse IgM HRP diluted 1:6000 (Invitrogen, USA). Plates were washed between incubations with 0.05% (v/v) Tween 20 in PBS. Plates were incubated with the substrate o-phenylenediamine and urea hydrogen peroxide for 15 min and reactions were stopped with 0.5 M citric acid. Absorbance was read at 450 nm using an automated multiwell plate reader (ELx800, BioTek, USA). Data are displayed as OD450 nm values and mean antibody titers. Titer was calculated as the maximum dilution of serum at which an OD450 reading of 0.3 or above was recorded (3 times reagent blank [all reagents except for serum] reading of 0.1). If an OD 450 nm reading of 3 times the reagent blank at the highest serum concentration was not achieved, a nominal titer value of 16 was assigned.

### 2.6 Peptide microarray studies

120 15-mer peptides derived from the scFv sequence, which has 252 amino acid residues in total (Edwardraja et al., 2010), with 14 residue overlap and 1 residue offset were synthesized by SPOT synthesis using standard Fmoc-based chemistry (Wenschuh et al., 2000). The peptides, with IgG and IgM protein controls, were then printed as spots in triplicate in an array format onto functionalized glass slides (Panse et al., 2004) (JPT, Germany). 1 μL of serum sample was diluted 1:125 in array buffer, 3% BSA, TBST: 50 mM Tris, 150 mM NaCl, 0.1% Tween 20 (Sigma Aldrich). Diluted samples were loaded onto the array slides positioned in a 96 well slide chamber and incubated for 1 h with shaking. Array slides were then washed three times in TBST and then incubated with detection antibody; Dylight 650 conjugated goat anti-mouse IgG at 1:1000 in array buffer (Thermo Fisher, USA) and incubated in the dark for 1 h with shaking at room temperature. Array slides were then finally washed three times in TBST and twice in MilliQ water. Slides were spun dry by centrifugation at 300 *g*. Slides were scanned with a Genepix 4000b scanner (Molecular Devices, USA) using the 635 nm laser (PMT gain: 700, 100%). Data were acquired using GenePix Pro 7.0 software. Routines in R were written to collect mean feature intensity from triplicate spots and, where coefficient of variance exceeded 50%, the closest duplicates were used, eliminating any significant outliers or feature artefacts.

### 2.7 Peptide synthesis

Epitope peptides for ELISA were produced as 15mers by standard solid phase synthesis employing fmoc chemistry including an N terminal Biotin and hexanoic acid linker (Pepceuticals Ltd, UK). The following sequences were synthesized; Peptide 53 Wt: STSYISDLWGQGTLV, Peptide 53 W113 A variant: STSYISDLAGQGTLV; Peptide 53 scrambled variant: LWTSDYLVGISTGSQ.

### 2.8 scFv model molecular dynamics simulation

A homology model for the human scFv, originally described by Edwardraja et al (Edwardraja et al., 2010) was generated using I-TASSER (Yang et al., 2015a). Molecular dynamics (MD) simulations were run in GROMACS 5.0.4. (Abraham et al., 2015) with the Amber 14 force field (Maier et al., 2015). The simulation protocol was as follows: (i) the protein was placed in a solvation box of minimum 12 Å around the protein, and counter-ions were added; (ii) energy minimisation was carried out and the system was thermalized at 300 K for 100 ps with constant pressure and 100 ps with pressure coupling (using the Parrinello-Rahman barostat), with constraints on the protein; (iii) unconstrained MD simulations were carried out using constant pressure. All simulations used a 10 Å cut-off for electrostatic and van der Waals interactions, the LINCS algorithm (Hess et al., 1997) for bond constraints, a 2 fs time step and periodic boundary conditions. Four simulated annealing simulations were carried out using pressure coupling from structures taken after 10, 20, 30 and 40 ns of MD simulations, with 2 ns 350 K/500 K temperature cycles including 100 ps heating/cooling periods. Root-mean-squared deviations (RMSD) for each residue relative to the structure at the start of the MD simulation was measured to assess the extent of unfolding, with similar results for the four annealing simulations.

### 2.9 Biophysical characterization of aggregates

Measurements of dynamic light scattering (DLS) was performed with a Malvern Zetasizer Nano ZS ZEN3600 (Malvern, Germany) equipped with a 633 nm laser. Each sample (70 μl) was measured in a Suprasil quartz cuvette (Hellma, Germany) with a path length of 3 mm and 200–2500 nm spectral range. Monomeric and heat stressed samples at 2 mg/ml in 10 mM sodium acetate, 15 mM sodium chloride were measured at 25 °C to determine the volume-based average protein particle diameter in solution.

Circular dichroism (CD) experiments were undertaken on a Chirascan spectrophotometer (Applied Photophysics, UK). Protein solutions of 50 μl volume at 2 mg/mL (sodium acetate buffer) were placed in a 1 mm pathlength quartz cell (Hellma, Germany) with Peltier temperature controlled stage ramping from 25 °C to 60 °C in 1 °C increments, with 1 min to equilibrate at each temperature. Spectra were recorded at 190–260 nm with an acquisition time of 2 s at 0.5 nm increments and normalized by subtraction with a corresponding buffer blank.

The diffusion coefficient and radius of hydration of each sample were determined using the Dynapro Nanostar system and DYAMICS software (Wyatt, USA), using the laser wavelength of 658 nm with a scattering angle of 90 °. 30 μl sample volumes were used in disposable cuvettes. The acquisition time was set at 5 s and 10 replicate collections were taken of each sample. Measurements were carried out at 25 and 40 °C, held isothermal for the duration of the experiment.

### 2.10 Statistical analysis

Statistical analyses were performed using the software Prism 7 package (Graphpad, USA). ELISA experiments were analysed by non-parametric 1 way ANOVA followed by the Tukey *post hoc* test to determine statistical significance of differences between treatment groups. For the peptide array data, a 2 way ANOVA with Holm-Siddak method was used to identify peptides that demonstrated significant difference in IgG binding between treatment groups (*P ≤ 0.05, **P ≤ 0.01, ***P ≤ 0.001, ****P ≤ 0.0001).

## 3 Results

### 3.1 Biophysical characterisation of scFv aggregate formation

A single chain Fv (scFv) was selected as an exemplar protein which aggregated readily at low temperatures, to avoid the risk of complete denaturation. The production of aggregates at lower, rather than higher, temperatures is more representative of aggregates formed during storage. We used a human scFv, originally described by Edwardraja *et al.*, as an ideal candidate for these studies, for two reasons. First, we have characterised its immunogenicity (Ratanji et al., 2016a, b) and the effects of surface charge on scFv aggregates (Austerberry et al., 2017); its aggregation behaviour is therefore well understood. Second, the protein is relatively small, making it practicable to cover the sequence by overlapping peptides in fine detail and facilitating molecular dynamics simulations (see below).

**Fig. 1:**
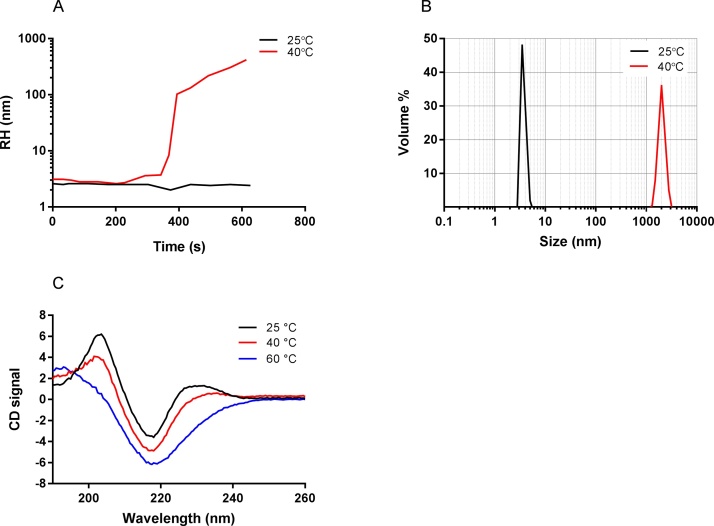
**Properties and kinetics of scFv aggregates.** A) 2 mg/ml scFv in 10 mM sodium acetate, 15 mM sodium chloride was incubated for 750 s at 25 °C (black) or 40 °C (red), and change in RH over time monitored by DLS, B) scFv at 1 mg/ml in PBS pH 7 was subjected to heat treatment for 25 min at 40 °C. The mean particle diameter (nm) was measured by DLS before (black) and after (red) the 40 °C incubation, C). CD spectra between 190–260 nm of 2 mg/mL scFv in 10 mM sodium acetate, 15 mM sodium chloride, subjected to heat ramping from 25 °C (black), 40 °C (red) and 60 °C (blue) in 1 °C increments with 1 min to equilibrate at each temperature, C. (For interpretation of the references to colour in this figure legend, the reader is referred to the web version of this article.)

Aggregation of the scFv was readily induced by a temperature increase from 25 to 40 °C; aggregation kinetics were monitored by DLS (Fig. 1A). An increase in the hydrodynamic radius (RH) from 3 nm to approximately 500 nm, indicative of the formation of aggregate particles, was seen within 5 min incubation at 40 °C. Aggregate sizes were analysed and were formed reproducibly within the 1000–3000 nm range after 25 min (Fig. 1B). To inspect the influence of thermal aggregation on protein folding, comparison of the secondary structure composition of the aggregate population at 25, 40 and 60 °C by CD showed that aggregates at 40 °C retained their secondary structure composition (Fig. 1C). At 25 and 40 °C the spectra indicate β-sheet content and at 60 °C the peaks in the signal have decayed, indicating loss of secondary structure (and thus tertiary structure), leaving only random β-coil species.

### 3.2 Characterization of antibody responses to scFv monomer and aggregate in a BALB/c mouse model

**Fig. 2:**
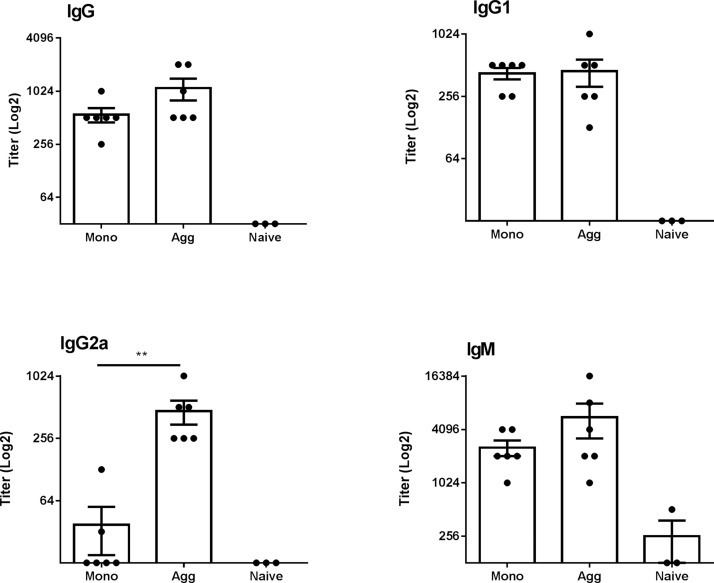
**Characterization of antibody responses to scFv monomer and aggregate preparations in BALB/c mice.** Mice (n = 6 per group) were immunized by i.p. injection with 250 μl of 1 mg/ml monomer or heat aggregated scFv on days 0, 7 and 14, and serum isolated on day 21. Doubling dilutions of serum samples from scFv monomer (Mono) and aggregate (Agg) immunized animals and negative control serum samples from naïve (untreated) mice (n = 3 per group) were analyzed against scFv monomer coated plates by ELISA for IgG, IgG1, IgG2a, anti-scFv antibody content. Ig Isotype and subclass data are displayed with respect to antibody titer (log2) calculated as the lowest serum dilution at which 3x the ELISA substrate blank OD450 nm reading was reached. Statistical significance of differences in antibody detection between all sera groups against each substrate was calculated using a 1-way ANOVA (**P ≤ 0.01).

We sought to identify whether the immunoprofiles of IgG and IgM antibody responses to scFv monomer and aggregate were different, using a well-established mouse model, which we have described previously (Ratanji et al., 2016a, b). BALB/c strain mice were immunized by i.p. injection with monomer or aggregated scFv on days 0, 7 and 14 (n = 6 per group). Sera were isolated on day 21. To compare the immune responses induced by monomer and aggregate, the serum levels of anti-scFv IgG, IgG1, IgG2a and IgM were analyzed by ELISA (Fig. 2). Data are displayed with respect to antibody titer (log_2_). As observed previously (Ratanji et al., 2016a, b), relatively high titers of anti-scFv IgG, IgG1 and IgM antibodies were detected in sera isolated from monomer- or aggregate-immunized mice. We did record a significant enhancement, however, in IgG2a antibody titer, increasing from ∼1 in 32 to 1 in 512- a 16-fold difference and characteristic of Th1-shewing, as observed previously (Ratanji et al., 2016a). We have shown previously that detection of anti-scFv IgM, IgG and subclasses is independent of whether the monomer or aggregate preparation is used as coating substrate in the ELISA (Ratanji et al., 2016a).

### 3.3 Immunoprofiling of monomer and aggregate antibody responses using a dedicated peptide microarray

**Fig. 3:**
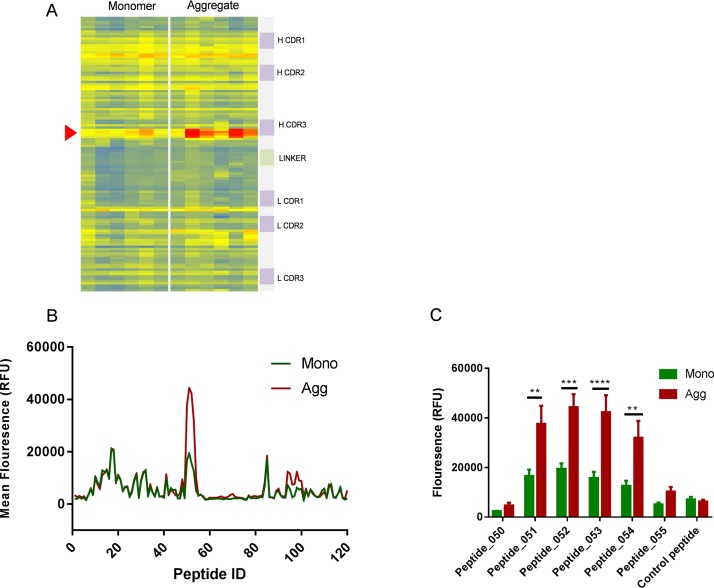
**Comparision of monomer and aggregate epitope signatures by peptide microarray.** A), Heat map of peptide microarray comprising 120 overlapping peptides originating from scFv heavy and light chains sequence incubated with individual serum (columns) from monomer and heat aggregated mouse immunization groups (n = 6). Heat map displays mean fluorescence intensity (increasing from blue to red) of each peptide detected by Dylight 650 labelled anti-mouse IgG antibody. Red arrow marks Peptide 53 region. B), Mean fluorescence profile of monomer and aggregate immunization groups), Box plot of mean fluorescence intensity at peptide region 50–55, displayed with overall mean and SEM. Human influenza hemagglutinin peptide used as control (sequence YPYDVPDYA). Statistical significance of differences in antibody binding peptides between sera groups was calculated using a 2-way ANOVA (**P0.01 ***P ≤ 0.001 ****P ≤ 0.0001). (For interpretation of the references to colour in this figure legend, the reader is referred to the web version of this article.)

Peptide mapping uses an array of overlapping peptides to define the distribution of regions of high antibody reactivity across a particular antigen. The small size of the scFv (252 amino acids) allowed us to cover the entire sequence with peptides which were incremented by a single residue. This corresponds to fine peptide mapping of the sequence which, we reasoned, allowed the best possible chance of identifying aggregation-specific neo-epitopes. A heatmap comparing total serum IgG profiles obtained using the peptide microarray, for mice administered with scFv monomer and aggregates, is presented in Fig. 3A.

The patterning of the epitope peptide binding was largely similar for mice within each group, and between monomer and aggregate treated groups (Fig. 3A). There were distinct hotspots of antibody recognition in the V_H_ domain, between the CDR1 and CDR2, and encompassing the CDR3. There was evidence of less antibody binding in the V_L_ domain compared to the V_H_, with epitopes between the CDR2 and CDR3 (Fig. 3A, B). Interestingly, these hotspots were not necessarily concentrated in the CDRs themselves, although they would be expected to be the sites of greatest sequence divergence between human and mouse heavy and light chain sequences. There were no obvious epitopes detected in the N terminus and the glycine-serine linker region. From a visual inspection of the heatmap, a sequence region that straddles the V_H_ domain CDR3 appeared to show the greatest difference in vigour of IgG binding between monomer and aggregate-derived sera. To confirm this observation, rolling averages of IgG levels for each peptide were determined from monomer and aggregate-treated animals. This region (peptide 51–54, sequence; DLGGSSSTSYISDLWGQGTLV) was confirmed to have substantially higher IgG levels for aggregate compared with monomer for four consecutive peptides (P ≤ 0.001, Fig. 3B and C). The epitope mapping data implies that there is selective increase in antibody titre to a specific epitope region of the scFv after challenge with aggregate species.

### 3.4 Comparison of epitope peptide binding by ELISA

**Fig. 4:**
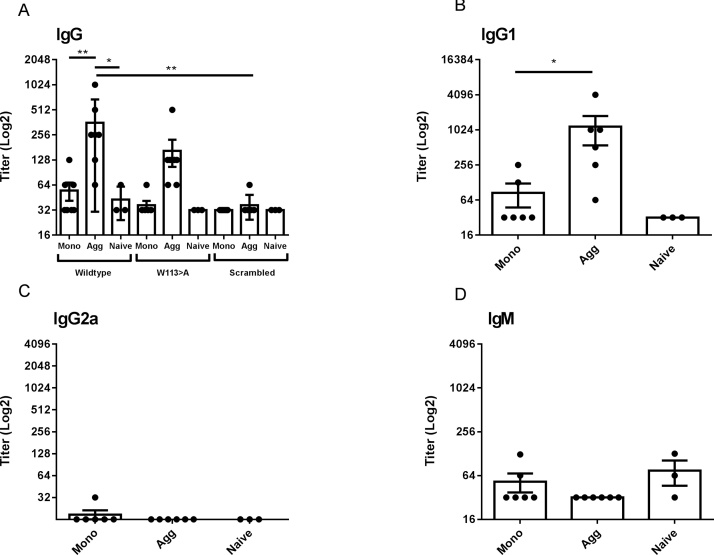
**Confirmation of epitope peptide binding by ELISA**. Serum samples from scFv monomer and aggregate immunized animals (n = 6) and negative control naıve serum samples (n = 3) were analyzed for total IgG titer to scFv biotinylated wildtype peptide 53, W113 A or scrambled control by, **A**. Antibody IgG subclass and isotype IgG1, IgG2a and IgM titer were determined from the serum groups against wildtype peptide 53 by ELISA, **B to D**. Data are displayed with respect to antibody titer (log2), calculated as the lowest serum dilution at which 3x the ELISA substrate blank OD450 nm reading was reached. Individual titers are displayed (●) with overall mean and SEM. Statistical significance of differences in antibody binding between all treatment groups against peptide were calculated using a 2-way ANOVA. *P ≤ 0.05 *** P ≤ 0.001.

We sought to confirm our observation that a locus of peptide sequences within the scFv reacted more strongly with IgG antibody derived from sera from aggregated-treated, as opposed to monomer-treated, mice. We developed an ELISA assay, using a biotinylated peptide substrate bound to the assay plate via immobilized neutravidin (see Materials and Methods). Wild type peptide 53 (sequence STSYISDLWGQGTLV) was selected to represent the epitope region and synthesized with biotin incorporated at the N-terminus. A W113 A variant (STSYISDLAGQGTLV) and scrambled variant (LWTSDYLVGISTGSQ) were also synthesized and tested. Peptide-coated plates were incubated with serum and analysed for mouse IgG, IgG subclass and IgM binding; the results are shown in Fig. 4. Total IgG was compared in sera derived from monomer and aggregate-inoculated mice that bound peptide 53 and peptide variants (Fig. 4A). The results confirmed that sera from aggregate-inoculated mice had a significantly increased IgG titer to peptide 53 compared to animals inoculated with monomer (P ≤ 0.001) or untreated, naive mice (P ≤ 0.001). Epitope sequence specificity was tested by substitution of the central Trp residue by Ala (W > A). In this case, the titer was reduced and, in the case of the scrambled sequence peptide, completely abolished (P ≤ 0.001).

Interestingly, specificity for the aggregate peptide was reflected in IgG1 subclass (Fig. 4B). IgG2a levels were not detectable, within the limits of assay sensitivity. There was no significant difference in IgM antibody response to the peptide epitope between treatment groups (Fig. 4D). Although there is a pronounced difference in reactivity of IgM, IgG and its subclasses towards the scFv protein in Fig. 2, it is clear that the data from the epitope peptide in Fig. 4 give a different profile.

### 3.5 Modelling and molecular dynamics simulations of scFv unfolding

**Fig. 5:**
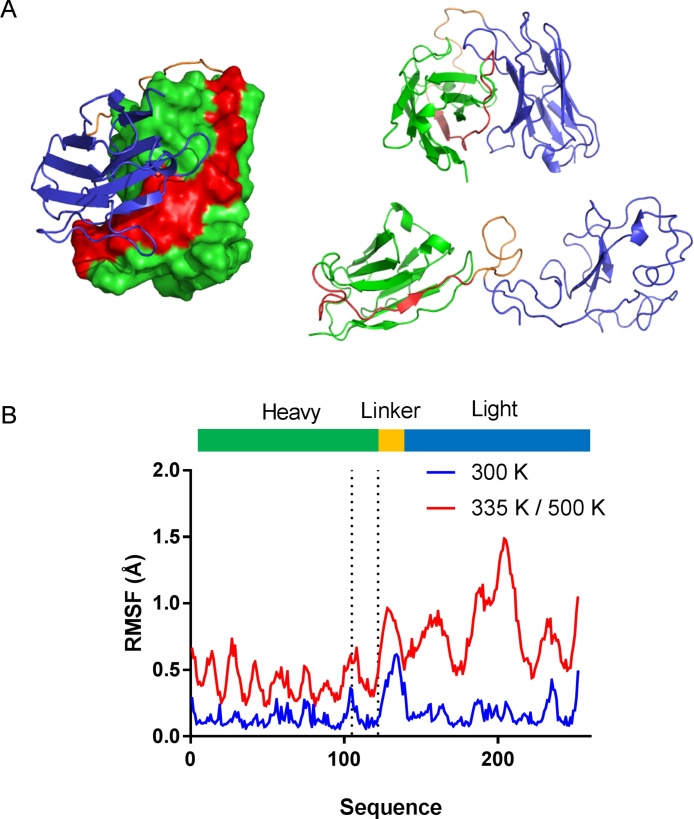
**Modelling and molecular dynamics simulations of scFv unfolding.** A. An scFv homology model (left) was created using I-TASSER (Yang et al., 2015b), along with a representative structures from the MD simulation (top) and simulated annealing (bottom). Peptide 53 epitope is highlighted in red. B. RMSD of individual amino acid residues was calculated from the MD and simulated annealing simulations. Heavy, light chain and linker domains are coloured in green, blue and orange respectively. The epitope peptide 53 region is indicated between the dotted lines. RMSD was calculated relative to the initial structure of the MD simulation, and mapped to the length of the scFv amino acid sequence. (For interpretation of the references to colour in this figure legend, the reader is referred to the web version of this article.)

We next attempted to interpret these results by reference to the predicted structure of scFv: a molecular model for the scFv was generated using I-TASSER, a template-based protein structure prediction software suite (Yang et al., 2015b). As anticipated, the results generated two immunoglobulin folds for the V_H_ and V_L_ domains. Peptide 53 was found to locate between the two domains on the more hydrophobic side of the V_H_ domain (Fig. 5A). Current theories of aggregate formation postulate the formation of partially unfolded species, which act as nucleation sites to trigger aggregate growth (Mahler et al., 2009). In order to examine differences between the folded and partially unfolded states of scFv, we performed molecular dynamics simulations and simulated annealing. At the lower temperature of 300 K, structural deviations are largely confined to the loop region between the V_H_ and V_L_ domains (Fig. 5B). During the simulated annealing, on the other hand, the V_L_ domain was more unfolded than the V_H_ domain. We suggest that partial unfolding of the scFv could result in greater exposure of the portion of the V_H_ domain which binds to V_L_, which includes the peptide 53 sequence. Transient exposure of this more hydrophobic portion of the protein could result in a species with increased potential to bind to other scFv molecules, possibly trapping partially denatured states within the aggregate.

## 4 Discussion

The motivation for this study was to examine whether aggregates can stimulate an ADA immunosignature which can be distinguished from that associated with the monomer. If so, this observation could be used to identify experimentally the proposition that the ADA response can adapt to epitopes that are formed or enhanced through aggregation. We selected an exemplar TPP scFv which aggregates under mild thermal stress and demonstrates differential immunogenicity towards aggregate species in the BALB/c mouse model. Aggregation causes a Th1 skewing in response, indicated by Th1 cytokine induction (IFNγ) and enhancement of IgG2a antibody isotype, akin to an immune response against repetitive antigens (Ratanji et al., 2016a). We set out to probe the epitope profile of the IgG antibody response and explore the distinction between monomer and aggregate recognition using peptide mapping. Here, we are employing this technology to ascertain whether we can identify a particular immunosignature associated with aggregation. This is probably more demanding than the conventional applications (e.g. vaccine research), as therapeutic proteins are generally weakly immunogenic, and the differences in immune response between native conformation, monomeric protein and its associated aggregates are likely to be subtle. Nevertheless, we were able to demonstrate that sera from aggregate-treated mice developed a stronger antibody response to a specific region identified from the peptide array, compared with monomer-treated mice and naïve (untreated) mice. Importantly, this was not a generalised increase in antibody responses to all peptides but was focused on one particular region of the molecule that encompasses the heavy chain CDR3. In response to aggregate challenge, there was a significantly enhanced IgG response to a focused epitope region of the protein. This was further supported by studies which demonstrated that it was a sequence-specific interaction: anti-sera did not react against a sequence-scrambled control peptide. IgG isotype studies were conducted and we found that the isotype reaction to the domain aggregate epitope was IgG1. As reported previously and reproduced here, responses to scFv protein aggregate are predominantly of IgG1 and IgG2a isotype in BALB/c (Ratanji et al., 2016a). Elevated IgG1 was detected by protein ELISA in both monomer- and aggregate-challenged mice whereas IgG2a was elevated only in aggregate challenge (Th1). We did not observe any IgG2a contribution to the dominant peptide epitope. The nature of the linear epitope mapping means, however, that we are only observing a fraction of the total IgG immune response: correspondence between IgG isotype responses to whole protein, on the one hand and to a single peptide antigen on the other, is not necessarily to be expected. We also note that IgM levels from aggregated-administered mice are not significantly different from monomer when detected using whole scFv (Fig. 2) or peptide (Fig. 4), indicating that T cell independent responses play little or no part in the selective response we observed to aggregated scFv.

It is useful to further highlight the technical limitations of the immunoassays employed here for measuring anti-sera specificity. Adsorption of a protein to a solid phase surface can result in denaturation, aggregation and variation in epitope presentation (Butler et al., 1992). Because of the undefined structure of a protein once adsorbed to an ELISA microtiter plate, discrimination of the specificity of anti-sera between monomer or aggregate species is confounded. Using peptide antigen covalently captured to a solid phase enhances both antigen presentation and density, enabling the detection of epitope-specific and low titer antibody populations that are otherwise difficult to resolve against the background of whole anti-sera to protein antigen. A caveat is that antibodies that are specific to purely conformational epitopes may not be efficiently captured by equivalent linear epitope peptides (Abbott et al., 2014; Forsström et al., 2015).

In this study a humanized scFv was used as an exemplar TPP; it was previously shown to be immunogenic in mice, therefore enabling the measurement of changes in the epitope immune profile upon aggregation. scFv domain constructs are relevant as potential therapeutic entities in themselves or as antigen recognition domains for larger therapeutic constructs (Boudousquie et al., 2017; Chan and Carter, 2010). Ig domain antibodies have been shown to be at particular risk of immunogenicity through exposure of cryptic epitopes not exposed in the native IgG structure (van Schie et al., 2015). scFvs are relatively flexible molecular constructs and the interdomain region is prone to exposure. Thermostability is impaired when compared to the parent Fab configuration or full IgG structure (Perchiacca and Tessier, 2012). This structural instability poses a risk of exposing epitopes through unfolding and subsequent aggregation (Maas et al., 2007). Indeed, we were able to rationalize the location of the epitope, through a molecular dynamics study, which suggests that this part of the V_H_ domain may be preferentially more exposed in the partially unfolded state. We suggest that partial unfolding of the scFv could result greater exposure of the portion of the V_H_ domain which binds to V_L_, which includes the peptide 53 sequence. Transient exposure of this more hydrophobic portion of the protein could result in a species with increased potential to bind to other scFv molecules, possibly trapping partially denatured states within the aggregate. V_L_ domains have been shown to be less thermostable by experimental measurement and tend to unfold first under thermal stress (Perchiacca and Tessier, 2012), exposing part of the V_H_ domain. We found that a tryptophan (W113) which is highly conserved in V_H_ immunoglobulin interfaces (Perchiacca et al., 2011), was at the core of the peptide epitope, and replacement with alanine reduced anti-sera recognition, highlighting the point that buried features can drive immunogenicity on exposure. To the best of our knowledge, this is the first report of the detailed mapping of an epitope enhanced through aggregation within a therapeutic protein, although the existence of such epitopes has been proposed in the past (Filipe et al., 2010). Cryptic epitopes are now of broader interest as targets to direct antibody therapy specifically against pathogenic aggregates in protein folding diseases (Phay et al., 2013; Sevigny et al., 2016).

Aggregation of therapeutic proteins is thought to be triggered by the association of partially unfolded forms (Kumar et al., 2011, 2010). Partial denaturation will lead to exposure of hydrophobic patches which would normally be buried in the fully folded structure. Once bound into the aggregate, these partially denatured states will remain. Methods for probing the secondary and tertiary structures of proteins in aggregates are limited, however. Some spectroscopic methods have been used- FTIR, for example (Joubert et al., 2011)- but they provide a measurement of the degree of preservation of the overall structure, rather than information on the conformation of individual parts. Some investigators have used amide hydrogen/deuterium (HD) exchange combined with mass spectrometry to monitor partial unfolding during aggregate formation (Arora et al., 2015). In this work we adopted a different approach to the problem, by seeking to identify whether antibodies which preferentially bind to aggregation-associated epitopes could be identified.

Here and elsewhere both animal and *in vitro* models have been used to exhaustively confirm aggregation results in enhanced immunogenicity to TPP (Ahmadi et al., 2015; Joubert et al., 2012). While aggregation is firmly accepted as a risk factor, the effect of aggregation alone on the current TPP products in use is less clear. There are several examples where detectable protein aggregation has been linked to immunogenicity in patients, such as human growth hormone and erythropoietin (Moussa et al., 2016; Sauerborn et al., 2010), but it is not yet linked to monoclonal antibodies with reported immunogenicity (van Brummelen et al., 2016; van Schouwenburg et al., 2013). All TPP preparations are likely to have some degree of protein aggregation (Ratanji et al., 2014) but it is likely that the size, amount and quality of the aggregates is crucial, as well as other product factors. The implication of our observations is that antibodies which react preferentially against immunogenic aggregates could exist in human sera, if sufficiently sensitive methods for their identification could be employed. Our study was conducted in mice, under controlled conditions. The expected variation of the immunoglobulin repertoire in human samples would likely complicate detection of specific antibodies by this method. However, from our studies it may be speculated that patients with high levels of ADAs also harbour antibodies which are specific for cryptic aggregation epitopes, a possibility which requires further experimental investigation.

## Figures and Tables

**Figure:**
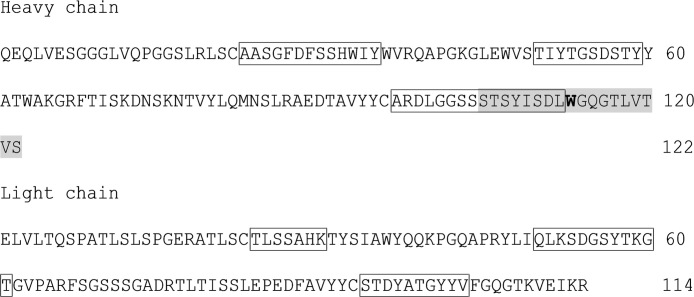


**Figure:**