# Supplementary material for: Identification of B cell epitopes enhanced by protein unfolding and aggregation
Source: Mol Immunol. 2019 Jan;105:181–9. doi: 10.1016/j.molimm.2018.11.020 (PMC6344229; doi:10.1016/j.molimm.2018.11.020)
Supplement: Supplementary file 1 [file mmc1.docx]

**Supplementary figures.**

**Figure S1** Amino acid sequence of anti-c-Met scFv heavy and light chain. The CDRs are indicated by boxes and labelled. Epitope peptide 53 indicated by grey shading with core tryptophan113 in bold.

**Figure S2** RMSD versus time (A) and RMSD along protein sequence (B) for the individual 300K and 350K/500K temperature cycling simulations.
